# Diagnosis of Graves’ Disease and Methimazole-Induced Lupus Erythematosus in an Adolescent Male During the COVID-19 Era: A Case Report

**DOI:** 10.7759/cureus.62023

**Published:** 2024-06-09

**Authors:** Tanya Kumar, Ashley M White

**Affiliations:** 1 Research and Development, Edward Via College of Osteopathic Medicine, Monroe, USA; 2 Pediatrics and Child Health, Willis Knighton Pediatric Healthcare Associates, Shreveport, USA

**Keywords:** adolescent covid-19, adolescent endocrinology, autoimmune disease and covid-19, covid-19, hyperthyroid, drug-induced-lupus, graves´disease

## Abstract

Graves’ disease is the most common form of hyperthyroidism in the pediatric population. Methimazole is the recommended regimen that is well-tolerated in most patients. Treatment with methimazole leading to drug-induced lupus erythematosus (DILE) is not well reported in the pediatric population, especially in the COVID-19 era. We present a case of a 14-year-old Caucasian male who presented with concerns of long COVID due to shortness of breath, hypertension, and fatigue. He was not noted to have significant weight loss, exophthalmos, or sleeping difficulties. He was followed by his general pediatrician, pediatric endocrinologist, cardiologist, and rheumatologist. Laboratory tests confirmed the diagnosis of Graves’ disease, and treatment was initiated with methimazole and atenolol. One month into treatment, the patient developed polyarthritis, urticarial rash, and difficulty with gait. Based on clinical suspicion and antibody panels, he was diagnosed with DILE secondary to treatment with methimazole. The patient was then started on a potassium iodide (Lugol) solution to promote the euthyroid state and proceed with total thyroidectomy. Post surgery, the patient developed hypothyroidism, which was managed with oral levothyroxine, to which the patient responded well. By discussing the clinical presentation and treatment of this patient, the goal is to raise awareness and increase clinical suspicion in diagnosing Graves’ and DILE in adolescents with upper respiratory presentations.

## Introduction

Graves’ disease (GD) is the most common cause of autoimmune hyperthyroidism in the United States, accounting for four of every five cases. While GD affects approximately one in 100 Americans [1], it is rare in children and adolescents, only affecting between 0.1 per 100,000 children and 3.0 per 100,000 adolescents per year [2]. Risk factors that predispose individuals to GD include female gender, age greater than 30 years, family history of GD or Hashimoto’s thyroiditis, or the presence of other autoimmune conditions [3]. Recently, numerous cases have suggested viral respiratory infections, including severe acute respiratory syndrome coronavirus 2 (SARS-CoV-2) infection, as a risk factor for developing new-onset autoimmune diseases.

In particular, long COVID often referred to as the post-acute phase of COVID-19 has been present in new-onset autoimmune conditions. Of the patients with COVID-19, approximately 10-30% of non-hospitalized patients and 50-70% of hospitalized patients have long COVID. The symptoms vary across age and organ systems, but common findings of hyperthyroidism such as chest pain, palpitations, cough, dyspnea, and fatigue are evident. Furthermore, preliminary studies suggest that in addition to the pulmonary manifestations of long COVID, patients may experience neurocognitive impairment and acute kidney injury [4].

In the pediatric population, GD may present with tachycardia, diarrhea, or increased appetite with or without weight loss. Other symptoms such as poor exercise tolerance, poor sleep, emotional lability, and moodiness are often misinterpreted as normal problems affecting school-aged children [5]. In some cases, hyperthyroidism has been associated with tall stature, rapid bone maturation, and exophthalmos. On laboratory analysis, thyroid-stimulating hormone receptor antibody (TSHR-Ab) has been found to have the strongest association with GD. The presence of the autoantibody increases thyroid activity leading to hyperplasia, increased hormone secretion, and possible thyrotoxicosis. Anti-thyroperoxide antibodies (TPOAb), when paired with anti-thyroglobulin antibodies (Anti-Tg) are more indicative of Hashimoto’s disease [6].

In terms of treatment, methimazole and propylthiouracil (PTU) are both considered first line. Methimazole and PTU inhibit thyroid hormone synthesis via interfering with thyroid peroxidase (TPO)-mediated iodination of tyrosine residues. PTU also inhibits peripheral conversion of thyroxine (T4) to triiodothyronine (T3) [2]. The most reported serious adverse effects of methimazole include agranulocytosis, hepatotoxicity, and hypothyroidism [7]. Methimazole is known to cause drug-induced lupus erythematosus (DILE); however, this is an infrequent adverse event, especially in the pediatric population, and it often develops weeks to months after the start of treatment.

To make a diagnosis of DILE, the following criteria are often referred to: (i) exposure to a drug that is suspected of inducing DILE for at least one month, (ii) symptoms of organ involvement such as myalgia, arthralgia, fevers, or rash, (iii) positive laboratory findings of anti-nuclear antibody (ANA), anti-histone in the absence of other antibody specificities such as anti-dsDNA, anti-Smith, (iv) no previous evidence of SLE, and (v) improvement of symptoms within weeks after discontinuation of drug [8,9].

In patients who could not tolerate methimazole, potassium iodide (KI) solutions were considered for long-term management of GD. However, studies have shown that extended use of KI worsens hyperthyroid symptoms experienced by patients and KI is now used as a bridging therapy to thyroidectomy to reduce anesthesia risk and thyroid storm. Patients must be euthyroid or have mild thyroid dysfunction at the time of thyroidectomy [10].

## Case presentation

A 14-year-old Caucasian male initially presented with a three-week duration of extreme shortness of breath, cough, congestion, and concerns about COVID-19. He was exposed to family members who had tested positive for COVID-19. He reported an inability to run and participate in sports due to shortness of breath and fatigue. He denied fever, chills, or body aches. He tested negative for COVID-19 and influenza at the time. His family history was significant for various auto-immune conditions. His mother was diagnosed with Hashimoto’s thyroiditis (age 18) and had a colon resection for multiple polyps (age 40). His father has a history of seven spontaneous pneumothoraces, potential Marfan syndrome (work-up incomplete), and hereditary angioedema. His older brother had one spontaneous pneumothorax requiring surgery. His paternal grandfather had a history of giant cell arteritis.

**Figure 1:**
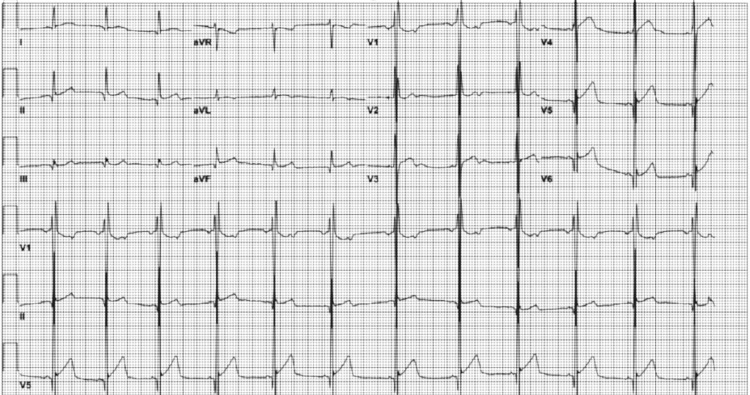
EKG findings on follow-up for shortness of breath eight months after symptom onset. EKG displays normal sinus rhythm (ventricular rate of 72 bpm, PR interval of 112 ms, QRS duration of 92 ms, and QT of 386 ms) and biventricular hypertrophy.

At a follow-up visit with his pediatrician eight months later, the patient still complained of extreme shortness of breath. Despite extra practice, his mile time went from seven minutes to nine minutes. Chest X-ray suggested perihilar infiltrates versus increased perihilar vascularity bilaterally. On physical examination, he presented with thyromegaly, elevated blood pressure (138/60), and 3+ patellar reflexes bilaterally. He did not have any leg swelling, palpitations, or tachycardia, but he reported worsening fatigue, lightheadedness, and urinary and vision changes. An EKG was ordered, revealing normal sinus rhythm and biventricular hypertrophy as seen in Figure 1. Cardiology was consulted and an echocardiogram was ordered. A transthoracic study was performed including 2D, M-mode, spectral, and color-flow. No significant cardiovascular findings were reported.

Complete metabolic panel (CMP), thyroid stimulating hormone (TSH), free T4, lipid panel, and erythrocyte sedimentation rate (ESR) were ordered. As seen in Table 1, all laboratory results were clinically insignificant, other than the low level of TSH and elevated level of free T4. The patient was advised to go to the emergency room for prompt treatment and was referred to a pediatric endocrinologist for further management. Repeat TSH and free T4 levels along with thyroperoxidase antibodies (anti-TPO) were ordered. The patient tested positive for anti-TPO, thyroglobulin, and thyroid-stimulating immunoglobulin as seen in Table 2.

**Table 1:** Initial laboratory tests ordered for the patient: complete metabolic panel and estimated sedimentation rate The patient's TSH measured below the reference range at <0.015 uIU/mL with a free T4 measurement greater than the reference range at 5.6  ng/dL. The estimated sedimentation rate is also increased in this patient. BUN: blood urea nitrogen; ALT: alanine transaminase; AST: aspartate aminotransferase; HDL: high-density lipoprotein; LDL: low-density lipoprotein; TSH: thyroid-stimulating hormone

Tests	Result	Units	Reference Range
Glucose	89	mg/dL	70-109
Potassium	4.8	mmol/dL	3.5-5.1
Sodium	140	mmol/dL	137-145
Chloride	105	mmol/dL	98-107
CO2	28	mmol/dL	21-32
BUN	14	mg/dL	7-20
Creatinine	0.48	mg/dL	0.5-0.9
Calcium	10.0	mg/dL	8.4 – 10.2
Alkaline Phosphatase	513	U/L	166 – 571
Total Bilirubin	0.7	mg/dL	0.2 – 1.3
ALT	44	U/L	0 – 50
AST	52	U/L	3 – 45
Total Protein	7.8	g/dL	6.3 – 8.2
Albumin	4.8	g/dL	3.5 – 5.0
Estimated Sedimentation Rate	42	mm/hr	0 – 15
LIPID PANEL			
Cholesterol	132	mg/dL	<200
Triglycerides	64	mg/dL	<150
HDL	48	mg/dL	≥60
LDL	71	mg/dL	<100
THYROID FUNCTION			
TSH	<0.015	uIU/mL	0.5 – 4.3
Free T4	5.6	ng/dL	0.9 – 1.6

**Table 2:** Thryoid-specific panel ordered for the patient On repeat testing, TSH and free T4 remained out of the normal reference range. The patient also tested positive for thyroid autoantibodies including thryoperoxidase and thyroid-stimulating immunoglobulin. Elevated thyroglobulin levels suggest active production of T3 and T4. TSH: thyroid stimulating hormone

Tests	Result	Units	Reference Range
TSH	<0.020	uIU/mL	0.4 – 5.0
Free T4	4.68	ng/dL	0.71 – 1.51
Thyroperoxidase Antibodies	>900	IU/mL	<9.0
Thyroglobulin	367	IU/mL	<4.0
Thyroid-stimulating immunoglobulin	4.8	IU/mL	<0.10

**Figure 2:**
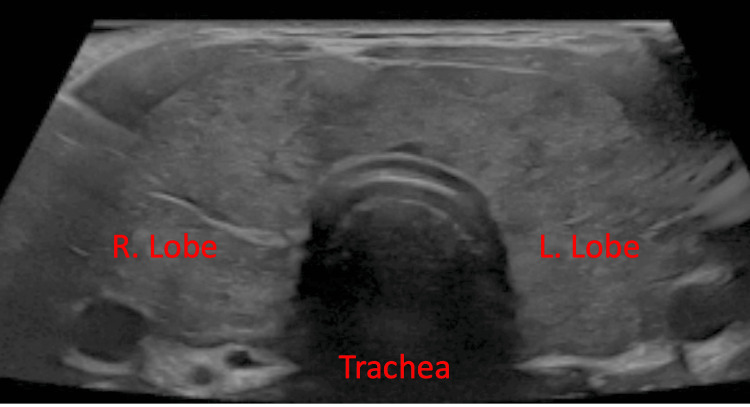
Thyroid ultrasound showing diffuse symmetric enlargement of the thyroid gland including isthmus. Right (R) thyroid gland measured 6 x 2.3 x 2.9 cm and left (L) thyroid gland measured 5.8 x 2.3 x 2 cm. Ultrasound findings also suggested bilateral reactive cervical lymph nodes and increased vascularity.

A Doppler sonographic evaluation of the soft tissue of the neck and thyroid was performed (Figure 2). Results suggested a diffuse symmetric enlargement of the thyroid gland including the isthmus. The thyroid gland was noted to have markedly hypoechoic heterogeneous spotty parenchymal patterns as well as increased vascularity on color Doppler. Based on the findings and potential for thyroid storm, a diagnosis of GD was determined, and the patient was started on empiric 10 mg methimazole thrice daily, and 50 mg atenolol as needed for rate control.

The patient adhered to the proper dosing protocol for methimazole but discontinued after approximately three weeks due to concerns of inability to walk, joint pain in the upper and lower extremities, and hives. The patient complained of morning joint stiffness lasting more than 30 minutes. He noticed an improvement in symptoms after discontinuation of methimazole. On physical examination, the patient had jitteriness, tachycardia, bounding pulses, limited lower extremity range of motion, bilateral knee pain, right hip pain, and swelling of the right knee joint. Complete blood count (CBC), CMP, ESR, C-reactive protein (CRP), ANA, perinuclear anti-neutrophil cytoplasmic antibodies (p-ANCA), anti-neutrophil cytoplasmic autoantibody (c-ANCA), anti-strep-O (ASO) titer, magnesium, and phosphorous levels were ordered. The patient was advised to hold methimazole and atenolol per his endocrinologist. As seen in Table 3, the patient had an elevated ESR and a positive ANA test.

**Table 3:** Autoimmune laboratory analysis to determine the cause of the patient's myalgia. The patient had an elevated estimated sedimentation rate suggesting acute inflammation. Positive antinuclear antibody result suggests an autoimmune process.

Tests	Result	Units	Reference Range
Estimated Sedimentation Rate	42	mm/hr	0 – 15
Antinuclear antibody	Positive		Negative
Perinuclear anti-neutrophil cytoplasmic antibodies	Negative		Negative
Anti-neutrophil cytoplasmic autoantibody	Negative		Negative
Anti-strep-O (ASO) titer	74	IU/mL	0 – 640
Magnesium	1.7	mg/dL	1.8 – 2.4
Phosphorous	5.9	mg/dL	3.5 – 5.3

Rheumatology was consulted for the migratory polyarthritis. ASO, human leukocyte antigen (HLA) B-27, and anti-histone antibody levels were ordered and no significant findings were evident, thereby ruling out reactive arthritis and suggesting DILE. Due to intolerance to methimazole, the pediatric endocrinologist prescribed six drops by mouth of Lugol’s 5% KI solution three times per day as short-term treatment. The patient was also switched from atenolol to propranolol. The patient followed the regimen and opted for extended treatment to ensure an euthyroid state. Due to intolerance of methimazole and the size of his goiter, ENT specialists were consulted for bilateral thyroidectomy as definitive treatment in place of radioactive iodine.

The patient underwent bilateral thyroidectomy. Post surgery, he was hypothyroid, requiring long-term treatment with levothyroxine. The patient was initially managed on 75 mcg and then switched to a 100 mcg tablet of levothyroxine every morning. He was advised to discontinue propranolol as his pulses were consistently in the low 50s. At the follow-up visit with the pediatric endocrinologist, labs revealed elevated TSH at 12.081 uIU/mL (reference range: 0.400 - 5.000 uIU/mL). The patient’s dose was adjusted to 125 mcg and then to 162.5 mcg every morning before breakfast to account for the increased TSH value. The patient is now euthyroid, with TSH and free T4 levels controlled within normal range. The patient’s calcium and parathyroid hormone (PTH) levels were assessed; calcium and PTH levels were normal.

## Discussion

COVID-19 has now been associated with the development of various endocrine disorders including GD. Our patient's symptoms of shortness of breath, dizziness, and fatigue, alongside his exposure to COVID-19, suggested an extended respiratory infection. One plausible explanation was that the COVID-19 virus can directly attach to the thyroid follicular and pituitary cells, thereby increasing the activity of the immune system and the quantity of antibodies present in an infected individual. Increased systemic inflammation can increase concentrations of interleukins such as IL-6, which inhibits thyroid-stimulating hormone secretion when secreted [11]. Mateu-Salat et al. describe two cases of thyroid disease within two months after the onset of COVID-19. In one of the cases, a 60-year-old female patient who had been diagnosed with GD 35 years prior and was in remission, presented with dyspnea and tested positive for SARS-CoV-2. However, in her case, imaging of the lung showed interstitial infiltrates in line with a diagnosis of COVID-19 pneumonia. After the patient’s symptoms improved, she had an onset of palpitations and fatigue, leading to a reassessment of thyroid function tests and thyroid antibodies which were positive [11]. The reactivation of thyroid disease in this patient could have been due to systemic inflammation. In patients with a strong family history of autoimmune conditions, such as in our patient, increased immune system activity and inflammatory markers such as interleukin (IL)-6 could increase the likelihood of autoimmune disorders. The 60-year-old’s presentation was very similar to that of our adolescent male; both of them presented with respiratory symptoms weeks to months before the onset of true palpitations, nervousness, and weakness.

An alternative to the systemic inflammation and direct attachment school of thought is the idea that antigen molecular mimicry cross-reaction could occur between the spike proteins of the virus and the TPO antigens. Antigen-presenting cells (APCs) and B cells allow the uptake of viral particles which are then presented to the CD4+ T cells by major histocompatibility complex (MHC) class II. The activated CD4+ cells lead to increased production of cytokines (tumor necrosis factor-alpha (TNF-α), IL-6, IL-17, Il-18, and IL-1ß), CD8+ T cell activation, and antibody production by B cells. Studies suggest a significant overlap exists between the amino acid sequences of the virus and the human tissue antigen. [12]. As a result, the antibodies produced against SARS-CoV-2 spike proteins interact with the host surface proteins leading to symptoms of tachycardia and chronic fatigue [11]. In patients that present with long COVID as in the case of our adolescent male, the body is susceptible to a greater range of diseases due to the increased activation of the immune system and constant fight against the virus [4]. Given that the patient’s maternal and paternal family history was significant for autoimmune conditions, his risk of developing an autoimmune disease was greater than in the population without a family history of autoimmune diseases.

In a study on patients admitted to the Department of Endocrinology at Children's Hospital of Nanjing Medical University, 68.7% of the pediatric patients (average age: 8.9 ± 2.9 years old) presented with an initial chief complaint of exophthalmos and 81.4% presented for neck swelling, 18.6% presented with palpitations, 11.3% presented with weight loss, and 10.3% presented with hyperhidrosis. Non-specific findings of weakness were present in about 22.1% of patients and 17.6% of the patients presented with concomitant respiratory tract infections [13]. In a report by Trinh et al., a 28-year-old female who developed GD after COVID-19 infection presented with complaints of palpitations and shortness of breath when exercising. On physical exam, the patient had painless thyromegaly, and lab analysis revealed similar findings to our patient [14].

We presented a case of an adolescent male with close to undetectable TSH and elevated free T4 levels. His initial complaints were shortness of breath, fatigue, and hypertension, which are mild findings of GD. Months later, before treatment with methimazole, the patient developed brisk (3+) reflexes bilaterally and significant palpitations. In contrast to the other cases that were previously mentioned, our patient had a more insidious onset of symptoms. He first presented with an upper respiratory infection suggestive of COVID-19 and progressed to more typical hyperthyroid symptoms over eight months. He did not display exophthalmos or hyperhidrosis. Similar reports of new-onset GD in the adolescent population exposed to COVID-19 infection or persistent viral illness in the years 2019 to the current day are sparse. Additionally, the presence of anti-TPO, thyroglobulin antibody, and thyroid-stimulating immunoglobulin together is an uncommon finding. The majority of patients test positive for one or two of the three antibodies in GD. Anti-TPO and thyroglobulin antibodies do not necessarily point toward GD diagnosis. 11.9% of young individuals can develop antibodies to thyroperoxidase but will present with normal thyroid function studies. For this reason, the presence of anti-TPO positivity does not provide any significant information in ruling out a diagnosis of GD [15].

Methimazole as a cause of DILE

The most common medications known to cause DILE include isoniazid, hydralazine, procainamide, TNF-α inhibitors, minocycline, and quinidine [16]. In adolescent females, the most frequent culprit reported is minocycline, a tetracycline antibiotic that binds to the bacterial 30s ribosomal subunit and inhibits protein synthesis, used in the treatment of acne vulgaris [17]. DILE often presents with symptoms of polyarthritis, swollen and tender joints, and pruritic rash. In one study on DILE, patients presented with either urticarial, malar, or vasculitis rash. Other symptoms included arthritic pains, fatigue, fever, and myalgias. P-ANCA was noted to be positive in 40% of the adolescents while ANA was positive in 80% of the adolescent study group [18]. Diagnosis is made based on clinical suspicion, as in the case of our patient. He presented with the typical asymmetric joint pains in three of his four extremities along with the urticarial rash. In the adult population, 40% of DILE patients present with fever, 5-25% present with photosensitivity or purpura, and up to 63% present with arthralgia [19]. The cutaneous manifestations are often considered non-specific findings. There are very few reports on the presentation of methimazole-induced lupus erythematosus in the age group of our patient to determine the similarity of presentation.

Methimazole is considered first-line therapy for hyperthyroidism in the adolescent population. PTU has a black box warning of hepatotoxicity and is recommended against in this age group. For patients in a thyroid ‘storm’ or crisis, secondary to untreated GD, infection, surgery, or radioactive iodine therapy, oral KI solutions can rapidly promote euthyroid states. KI should be given to patients at least one to two weeks before thyroidectomy to normalize free T3 levels and reduce the risk of thyroid storm [20]. In the pediatric and adolescent age groups, total thyroidectomy is deemed the definitive treatment for those with contraindications to radioactive iodine therapy, or for those with large goiters. Subtotal thyroidectomy can potentially cause recurrent hyperthyroidism. In our patient’s case, the patient’s goiter enlarged over the course of his treatment with methimazole and the transition to KI before surgery. KI was used to promote the euthyroid state and reduce preoperative bleeding. However, the patient's goiter rapidly increased in size despite KI treatment which suggested that he was not a candidate for radioactive iodine therapy and required emergent total thyroidectomy.

## Conclusions

This report discusses an adolescent male diagnosed with GD during the COVID-19 pandemic who was treated with methimazole leading to DILE. His clinical presentation was unlike those of other adolescents in his age group, likely due to concomitant long COVID infection. Since long COVID infections present with symptoms mimicking autoimmune conditions, further evaluation should be performed on these patients to differentiate the two states for research and clinical practice. To enhance knowledge about the diagnosis of GD in adolescents during COVID-19, it is proposed to maintain a registry of similar cases of individuals who were predisposed to autoimmune conditions, exposed to COVID-19, and presented with long COVID. Furthermore, DILE caused by methimazole is a rare phenomenon that should be further studied to inform pediatricians concerning the high index of clinical suspicion needed to diagnose and treat patients resistant to anti-thyroid therapy promptly. Furthermore, studies should be performed globally to obtain more data on associations between new-onset autoimmune disorders and SARS-CoV-2 exposure.
